# Effect of body contour changes on the setup and dosimetric accuracy of radiotherapy after cervical cancer surgery

**DOI:** 10.3389/fonc.2024.1392741

**Published:** 2024-09-02

**Authors:** Yu Li, Wuji Sun, Shilong Liu, Wenming Xia, Xu Yang, Libo Wang, Chao Ge, Kunzhi Chen, Yinghua Shi, Huidong Wang

**Affiliations:** ^1^ Department of Radiation Oncology & Therapy, The First Hospital of Jilin University, Changchun, China; ^2^ Jilin Provincial Key Laboratory of Radiation Oncology & Therapy, Department of Radiation Oncology & Therapy, The First Hospital of Jilin University, Changchun, China; ^3^ NHC Key Laboratory of Radiobiology, School of Public Health, Jilin University, Changchun, China

**Keywords:** cervical cancer, volumetric modulated arc therapy, body contour change, delivery accuracy, setup accuracy

## Abstract

**Purpose:**

The body contour of patients with cervical cancer is prone to change between radiotherapy sessions. This study aimed to investigate the effect of body contour changes on the setup and dosimetric accuracy of radiotherapy.

**Methods:**

15 patients with cervical cancer after surgery were randomly selected for retrospective analysis. The body contours on the once-per-week cone-beam computed tomography (CBCT) were registered to the planning CT (pCT) for subsequent evaluation. A body contour conformity index (CI_body_) was defined to quantify the variation of body changes. The body volume measured by CBCT was collected, and its relative difference in reference with the first CBCT was calculated and denoted by ΔV_n_. The relative setup errors, denoted by ΔSE_LR_, ΔSE_AP_, ΔSE_SI_, and ΔSE_vec_ for left–right, anterior–posterior, superior–inferior, and vectorial shifts, respectively, were defined as the difference in measured setup errors between the reference and following CBCTs. The planned dose was calculated on the basis of virtual CT generated from CBCT and pCT by altering the CT body contour to fit the body on CBCT without deformable registration. The correlations between body contour changes and relative setup errors as well as dosimetric parameters were evaluated using Spearman’s correlation coefficient *r_s_*.

**Results:**

CI_body_ was found to be negatively correlated with the superior–inferior and vectorial relative setup errors ΔSE_SI_ (*r_s_* = −0.448, *p* = 0.001) and ΔSE_vec_ (*r_s_* = −0.387, *p* = 0.002), and no significant correlation was found between relative setup errors and ΔV_n_. Moreover, ΔV_n_ was negatively correlated with ΔD_2_ (*r_s_* = −0.829, *p* < 0.001), ΔD_98_ (*r_s_* = −0.797, *p* < 0.001), and ΔTV_PIV_ (*r_s_* = −0.819, *p* < 0.001). ΔD_2_, ΔD_98_, and ΔTV_PIV_ were negatively correlated with ΔV_n_ (*p* < 0.005). No correlation was found for other examined dosimetric parameters.

**Conclusion:**

The body contour change of patients could be associated with the setup variability. The effect of body contour changes on dose distribution is minimal. The extent of body change could be used as a metric for radiation therapists to estimate the setup errors.

## 1 Introduction

Cervical cancer is the fourth most common female cancer worldwide, with 60% of patients being diagnosed under the age of 50 years (1, 2). Radiotherapy for cervical cancer after radical surgery reduces the risk of local recurrence (3, 4). Treatment planning is typically performed on the basis of planning computed tomography (pCT) dataset of patients. During the course of radiation therapy, anatomy changes in patients are inevitable due to weight change, patient setup, and bladder and rectal fillings (2, 5–7). The effects of dietary control, drug effects, and toxic reactions to radiotherapy predispose to changes in body shape, especially in the abdomen. Consequently, the body contour is expected to deviate on each treatment fraction, as the setup tattoos could move with respect to the internal anatomy, which could lead to an increase in the setup error without daily imaging guidance (8–11). However, to the authors’ knowledge, the effect of body contour changes on the setup error is yet to be investigated.

Body contour changes could also cause variations in dose projection with deviated beam path and entry angle, which could be substantial for intensity-modulated radiotherapy characterized by high dose conformality and steep gradients (12). In particular, when using volumetric modulated arc therapy (VMAT) involving simultaneous modulation of the gantry speed, dose rate, and multi-leaf collimators, the effect of body contour changes on the dosimetric outcome is difficult to evaluate, and deviations between planned and delivered doses are complicated (13, 14). Replanning may be necessary with the body contour changes exceeding tolerance, and the examination of pre-treatment cone-beam CT (CBCT) is the primary tool for such judgements (15–18). The dose deviation could be quantitatively assessed by calculating the dose distribution on the basis of CBCT–CT registration (19–21). However, this method is not quite practical because the process is time-consuming and the imaging quality of CBCT could limit the calculation accuracy.

Several studies have investigated the effect of body contour change on the dose distribution and suggested various efficient methods for assessment. Weppler et al. proposed a body contour change threshold of 1.5 cm for replanning for head and neck cancer (22). Sun et al. proposed several rules of thumb for dose percentage change and isodose line shift caused by body contour change for prostate and head and neck cancer (12). However, few studies have examined this issue on the basis of patients’ body changes throughout the treatment course. Besides, for cervical cancer after surgery, where the patient anatomy is prone to change, the dose variation caused by body contour changes is worth investigating. In addition, a quantitative metric could be advantageous for reliable assessment.

Body contour change could significantly increase patient positioning variability. With proper imaging-guided positioning, the resulting dose distribution could generally remain within an acceptable range with the presence of body contour and size changes. However, the dosimetric accuracy without daily imaging guidance could be impaired with increasing body changes and corresponding setup errors. This study aimed to investigate the correlation of body contour change and the setup and dosimetric accuracy and provide an efficient metric for treatment accuracy.

## 2 Materials and methods

### 2.1 Patients

A total of 15 patients with cervical cancer, who received VMAT radiotherapy after surgery in The First Hospital of Jilin University from 2019 to 2022, were randomly selected for this study. **Table 1** summarizes the information of the patients. The height and weight of each patient was collected on the day of CT simulation to calculate the body mass index (BMI; the body mass divided by the square of the body height).

**Table 1:** Patients’ characteristics.

Patient No.	Age (years)	Stage	BMI (kg/m^2^)
1	54	II	22.54
2	35	IIA	36.58
3	39	IB1	22.60
4	63	IIIB	26.78
5	59	IB2	20.57
6	51	IIA1	27.47
7	34	IV	22.04
8	42	II	23.94
9	64	II	25.64
10	61	IB	29.30
11	61	IB2	26.95
12	43	IB2	27.48
13	57	IB1	19.56
14	81	IB1	25.08
15	60	II	20.03

Patients were immobilized in supine position with thermoplastic masks (Klarity Medical & Equipment Co. Ltd., Guangzhou, China). The pCTs were acquired with 5 mm slice thickness using the Philips Brilliance Big Bore CT scanner (Philips Healthcare, Cleveland, OH) and then transmitted to the Eclipse treatment planning system (TPS) version 15.6 (Varian Medical Systems, Palo Alto, CA, USA).

### 2.2 Target definition and treatment planning

According to the Radiation Therapy Oncology Group 0418 protocol (23), the clinical target volume (CTV), organs at risk (OARs; bladder, rectum, small bowel, and left and right femoral heads) and body were delineated on the pCT. The planning target volume (PTV) was obtained by adding an isotropic 5 mm margin to the CTV. The PTV was prescribed with a dose of 50 Gy in 25 fractions. Dual-arc VMAT plans using 6 MV photon beam were generated in the Eclipse TPS. The dose distribution was calculated using AcurosXB version 15.6 algorithm with a grid size of 2.5 mm. All plans were normalized to deliver 100% of the prescribed dose to 95% of the PTV. One TrueBeam linear accelerator (Varian Medical Systems, Palo Alto, CA, USA) was used to treat all patients.

### 2.3 Body contour change and setup errors

All patients underwent pre-treatment CBCT in the first fraction and at least once in each following week. The CBCT images were acquired using the clinical pelvis protocol (125 kV, 1080 mAs, half-fan mode, full trajectory, 88 slices with 2 mm slice thickness, 47 cm field of view) on a Varian on-board CBCT. If the obtained setup error exceeds or nears the clinical tolerance in the previous CBCT, CBCT would also be performed in the subsequent fraction, and the localization marks would be revised when applicable. In fractions with no CBCT taken, patient setup would be based on localization marks and reference couch positions. If the deviation of the couch position is beyond tolerance, patient repositioning and CBCT would be performed. A total of 77 CBCT images were collected (five CBCTs for thirteen patients and six CBCTs for two patients). CBCT–CT rigid registrations were performed on the basis of bony anatomy, and manual adjustments may be involved if necessary. The resulting treatment couch shift was applied automatically. The couch shift obtained in the first CBCT for each patient was defined as the reference setup error. The relative setup errors, denoted by ΔSE_LR_, ΔSE_AP_, ΔSE_SI_, and ΔSE_vec_ for left–right, anterior–posterior, superior–inferior, and vectorial shifts, respectively, were defined as the difference between the reference and the following setup errors obtained in CBCTs to avoid potential errors from differences between simulation and treatment units as well as operation therapists. In addition, the interval days between CT and CBCTs were collected.

**Figure 1:**
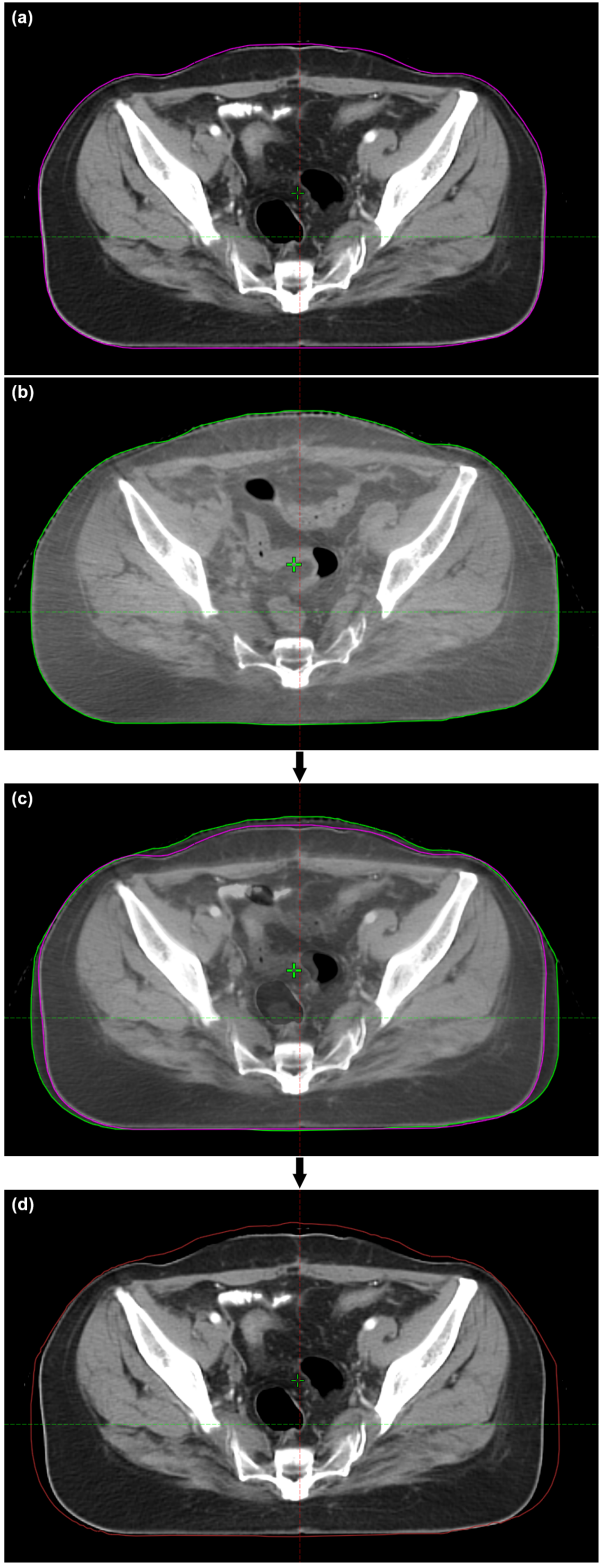
Demonstration of vCT generation for a patient. **(A)** pCT; **(B)** CBCT; **(C)** CBCT–pCT registration; **(D)** vCT. The CT values in the added area within the updated body contour of vCT are set as 0 HU.


CBCT images were imported into TPS, and the body volume of each CBCT (V_body_) was extracted. As shown in **Figure 1**, each pair of CBCT and pCT datasets was rigidly registered. The body conformity index (CI_body_) was defined as follows to quantify the degree of body contour change in each CBCT (n ≥ 2):

(1) $${\text{CI}}_{\text{body}}\text{ = }\frac{{\text{V}}_{\text{n}\cap 1}^{2}}{{\text{V}}_{\text{n}}{\;\times \text{ V}}_{1}}$$

where V_n∩1_ is the overlapping body volume in CBCT_1_ and CBCT_n_, and V_1_ and V_n_ are the body volumes in CBCT_1_ and CBCT_n_, respectively.

The differences in body volumes between CBCT_1_ and the following CBCT_n_, denoted by ΔV_n_, were calculated as follows:


(2) $$\Delta \text{Vn}\text{ = }{\text{V}}_{\text{n}}{\;\times \text{ V}}_{1}$$

which was used as a surrogate of body size changes. The correlations between relative setup errors and ΔV_n_ as well as CI_body_ were evaluated.

### 2.4 Body contour change and dosimetric accuracy

As the CBCT field of view is limited, only the overlapping body contour was assessed. The body contour in CBCT was copied into pCT, and the volume outside this contour was assigned with a CT number of −999 Hounsfield units (HU). The gap between the original and the new body contours in pCT were assigned with 0 HU for simplicity. Although in reality, this part could mostly be adipose tissue (mass density of ~0.9 g/cm^3^) with a typical CT number ranging from −190 to −30 HU (24), it could not substantially affect the dose distribution. The overlapping partin pCT and CBCT remained unchanged, including the PTV and OARs. Deformed registration of the patient anatomy was not used in this study to exclude confounding factors for the dosimetric variation caused by body contour changes. The resulting dataset was defined as the virtual CT (vCT_n_; n represents the order of CBCT). In order to eliminate the impact of the systematic deviation between CT and CBCT operations, the vCT_1_ was used as the reference image, which was later compared with each subsequent vCT_n_ for the dosimetric evaluation.

The original treatment plan was copied onto each vCT, and the plan doses were recalculated with the same monitor units. The variations of dosimetric parameters with ΔV_n_ and CI_body_ were evaluated, including CI, D_2_, D_98_, TV_PIV_, small bowel V_40Gy_, rectum V_30Gy_, bladder V_45Gy_, and femoral head V_30Gy_. The conformity index (CI) was calculated using Paddick’s formula as follows (25, 26):


(3) $$\text{CI = }\frac{{\text{TV}}_{\text{PIV}}^{2}}{\text{TV}\;\times \;\text{PIV}}$$

where TV indicates the PTV volume, PIV indicates the prescription isodose volume, and TV_PIV_ indicates the target volume within the PIV.

### 2.5 Statistical analysis

Spearman’s rank correlation coefficient (*r*_s_) was used to evaluate possible correlations of CI_body_ and ΔV_n_ with setup errors, treatment time, BMI, and dosimetric parameters. *p* < 0.05 was defined as statistically significant. |*r*_s_| ≥ 0.7 was considered a strong correlation, 0.7 > |*r*_s_| ≥ 0.5 was considered a moderate correlation, 0.5 > |*r*_s_| ≥ 0.3 was considered a week correlation, and |*r*_s_| < 0.3 was considered no correlation. All statistical analyses were performed using IBM SPSS Statistics 26.0 software (IBM Corporation, Armonk, NY).

## 3 Results

**Figure 2:**
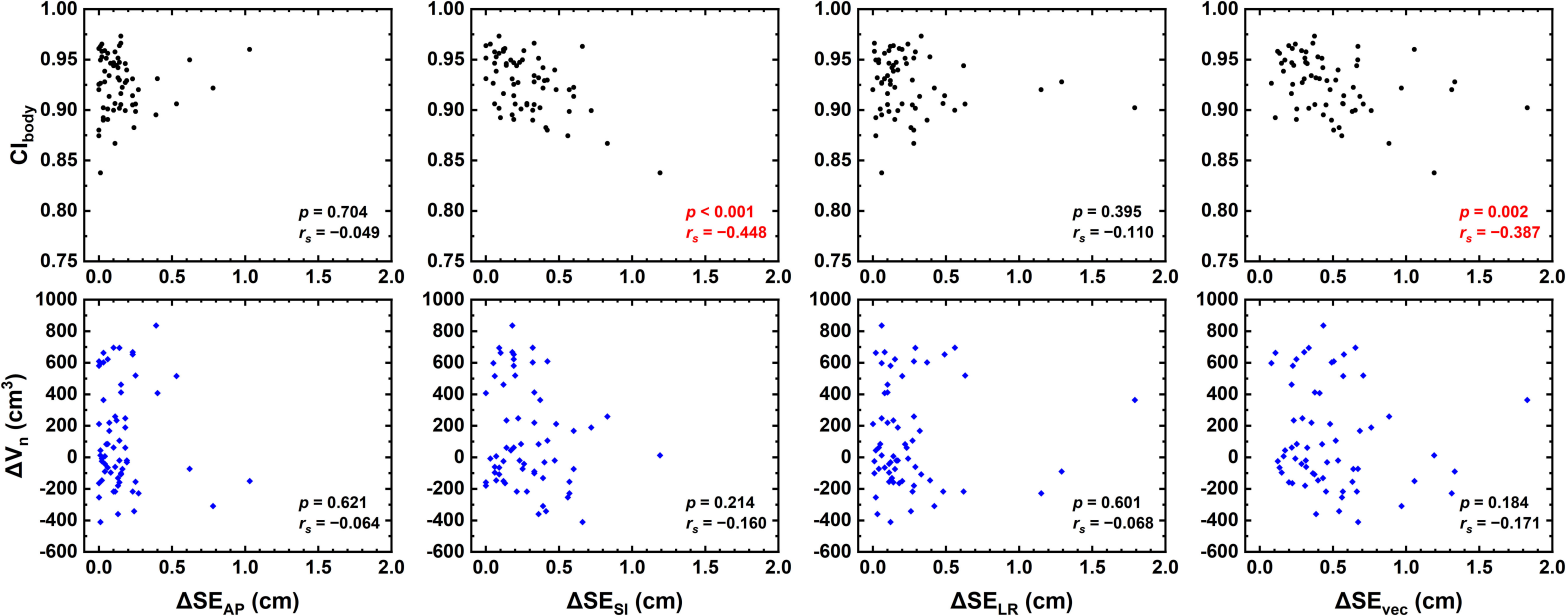
Correlation between relative setup errors (ΔSE_LR_, ΔSE_AP_, ΔSE_SI_, and ΔSE_vec_) and CI_body_ as well as ΔV_n_.

**Figure 2** demonstrates the correlation between relative setup errors and CI_body_ as well as ΔV_n_. ΔSE_SI_ (*p* = 0.001, *r*_s_ = −0.448) and ΔSE_vec_ (*p* = 0.002, *r*_s_ = −0.387) were found to be negatively correlated with CI_body_, and no correlation was found for ΔSE_LR_ nor ΔSE_AP_ (*p* > 0.05). No significant correlation was found with ΔV_n_.

**Figure 3:**
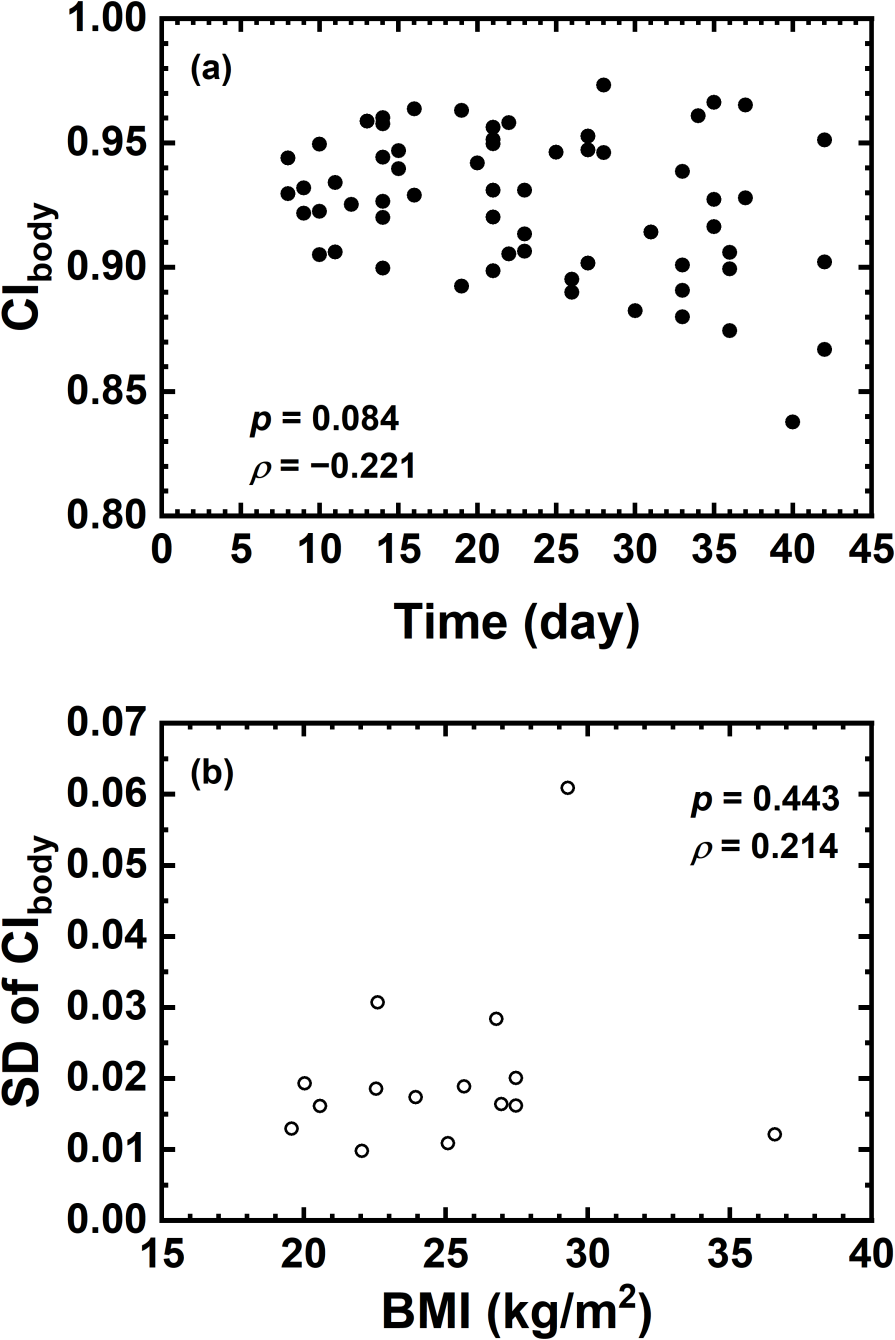
**(A)** Correlation between CI_body_ and Time (day); **(B)** correlation between standard deviation of CI_body_ and BMI.

**Figure 3** demonstrates that no significant correlation was found between the variation of CI_body_ and treatment time nor between the standard deviation of CI_body_ and BMI.

**Figure 4:**
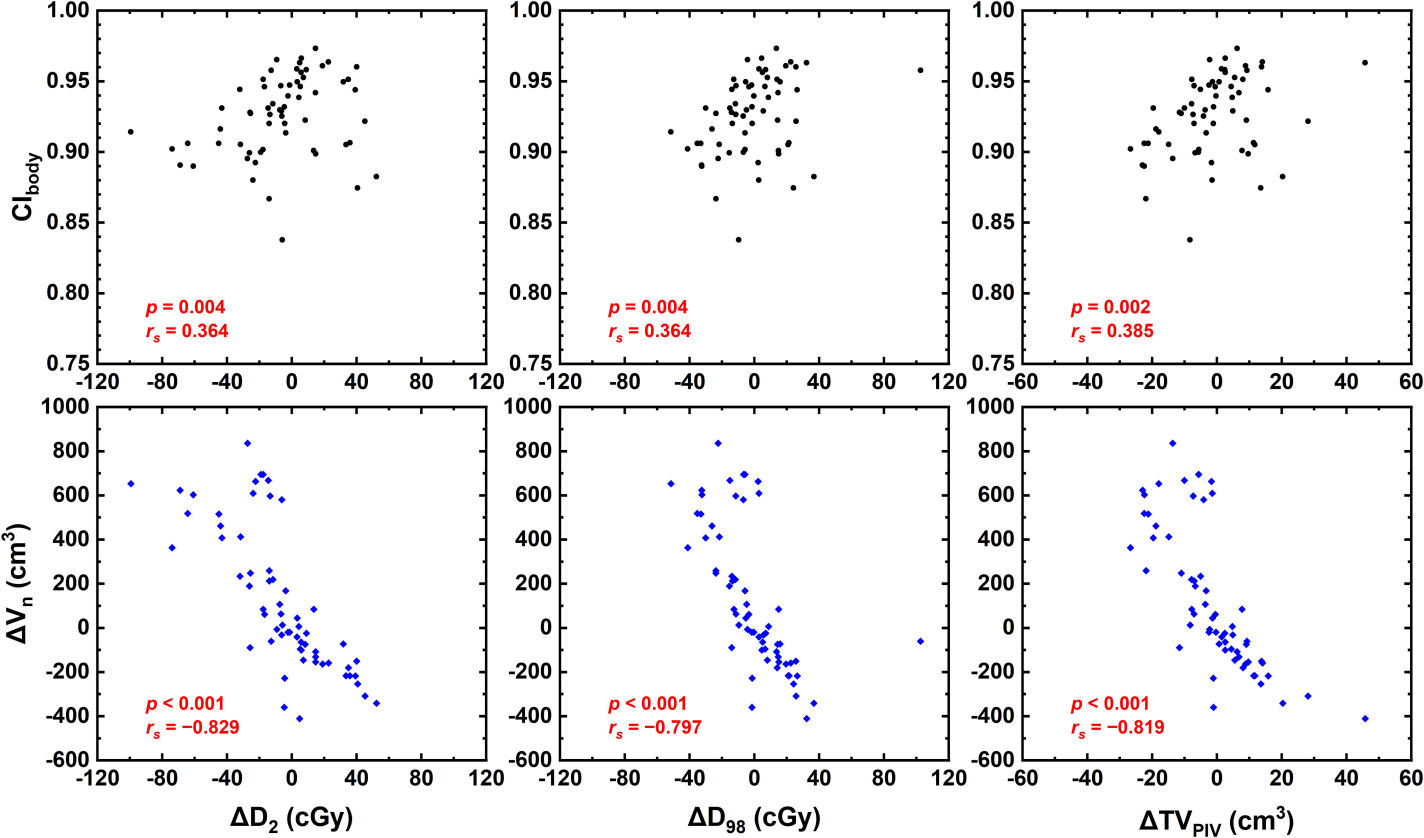
Correlations between dosmetric parameters CI_body_ and ΔV_n_.

**Figure 4** shows that CI_body_ and ΔV_n_ were significantly correlated with ΔD_2_, ΔD_98_, and ΔTV_PIV_ (*p* < 0.005). Strong or near-strong correlations were found for ΔV_n_ with these parameters, while weak correlations were found for CI_body_. No correlations were found for the other examined dosimetric parameters, including CI, HI, and OAR dose-volume parameters of interest.

## 4 Discussion

During the treatment of patients with cervical cancer, changes in body contour is expected in each treatment fraction. This study primarily focused on evaluating the effect of body contour changes on the setup errors and dosimetric accuracy in cervical cancer radiotherapy, providing parameters for prompt assessment of treatment accuracy.

CI_body_ and ΔV_n_ were defined to measure the discrepancy in the patients’ body contour to quantitatively assess the body contour change. As mentioned above, the first CBCT of each patient was used as the reference dataset for the assessment of setup errors rather than the pCT. The aim of using the first CBCT as the reference is to exclude the potential errors brought by differences between CT simulations, treatment units and operation staff. Therefore, the relative setup errors, i.e., ΔSE, were calculated for subsequent analyses for the correlation between setup errors and CI_body_ as well as ΔV_n_.

As shown in **Figure 2**, the relative setup errors ΔSE_SI_ and ΔSE_vec_ were negatively correlated with CI_body_. Patni et al. measured higher SI setup variations than AP and LR variations for uterine and cervical cancer (17), in line with the present data. By comparison, no correlation was found with ΔV_n_, indicating that CI_body_ is a better metric to access patient setup variability. Patients with large setup variations might not have large body contour changes, but patients with out-of-tolerance CI_body_ would potentially possess a greater risk of larger setup variations. Since daily CBCT is time-consuming and may not be realistic in practice, CI_body_ could serve as an assistant metric for the consideration of CBCT schedules.

Regarding the dose distribution in this study, ΔV_n_ was strongly or near strongly correlated with ΔD_2_, ΔD_98_, and ΔTV_PIV_, which are essentially the parameters susceptible to the beam attenuation variation caused by increased or decreased body size. However, the CI, HI, and all examined OAR dosimetric parameters showed no correlation with ΔV_n_, mainly because the dose calculation was performed on the basis of vCT generated with CBCT–pCT rigid registration with only body contour updated to isolate the effect of body contour changes. The internal changes in the patient anatomy would be a greater influence factor on the dosimetric distribution. Considering the patient anatomy change was not accounted for, the dosimetric change could solely depend on the body contour change, which is also the reason that no correlation between CI_body_ and most dosimetric parameters was found. Although CI_body_ was weakly correlated with ΔD_2_, ΔD_98_, and ΔTV_PIV_, it is hardly a meaningful correlation considering that CI_body_ could not reflect whether the body volume increases or decreases. Besides, the target region of patients with cervical cancer is relatively large, whereas the CBCT field of view is limited and hence could not fully cover the whole target area and OARs. Despite that the current results implied that the body contour changes may not cause substantial dosimeric variation, the patient setup variability could be increased for patients with steep changes in the body contour and body size. The increased risk of worsening dose distribution is not negligible, especially without daily CBCT.

The effect of changes in body contour, body size, or body weight on the dose distribution have been investigated in a number of studies (27–31). Miguel et al. (27) studied the effect of body change of patients with prostate cancer on the planned dose and found that the difference between planned and actual doses could be more than 5% when the difference between the anterior and lateral contour of patients exceeded 1.7 cm. Sasaki et al. (28) investigated the dosimetric effect of body contour changes in patients with cervical cancer and suggested that re-planning is rarely necessary with the body contour manually reduced by 1 cm in the front, whereas the variation became significant with the body contour reduced by 1 cm in all directions except the back, implying that dose distribution is affected by body size. The simulated variations of body contour and body size differed from the variations based on CBCTs in this study, but the results are in agreement. D’Souza et al. (29) evaluated the dosimetric effects due to changes in external body surface during the treatment course for prostate and head and neck cancers and proposed a 3.7%–5.2% change in the plan maximum dose per centimeter change in path length to isocenter. The body contours in the above two studies were either reduced or increased globally, which is rarely the case in clinical practice. In the present study, the body contour changes were extracted from patient CBCTs. The variation of body contour is complicated, and it may include simultaneous expansion and shrinkage. Therefore, the dosimetric effect was not considerably substantial as in previous studies.

Although the results in the present study suggested that the conformity of body contour throughout the treatment course may not significantly affect the dose distribution as long as the body size did not change drastically, it may not be the case without pre-treatment CBCT. The setup error variability could rise with increased discrepancy in the body contour, which could lead to a potentially greater difference in the planned and delivered doses. Based on the setup and dosimetric effect of body contour changes, radiation therapists should pay further attention to the change in body contour through pre-treatment CBCT in clinical practice. The extent of body contour changes defined by CI_body_ and ΔV_n_ could be used as reference parameters for radiation therapists to provide feedback to physicians on whether replanning is required. Actionable cutoff values for the metrics would be practical for this method to be applied in practice. Considering that the clinical protocol varies among treatment centers, the adopted cutoff could be decided individually.

One of the limitations in this study is that the BMI of each patient was only recorded prior to treatment but not in each fraction. The initial BMI is analyzed with the standard deviation of CI_body_ for the predictability of body changes. The present result indicated that the initial BMI could not serve as a predictor of body contour changes during the treatment course. BMI or body weight in each treatment fraction could be a potential indicator for body contour changes. Another limitation is that, as mentioned above, the current dosimetric evaluation was performed on the basis of vCT with only body contour changed within CBCTs. The patient anatomy was kept unchanged to isolate the effect of body contour changes. Deformable registration of patient anatomy in the vCT could potentially provide different dosimetric results (19–21), which is not within the scope of this study.

## 5 Conclusions

CI_body_ and ΔV_n_ were proposed to quantitatively assess the extent of body contour changes. CI_body_ showed moderate correlation with setup errors in the SI direction, and ΔV_n_ was strongly correlated with dosimetric changes including ΔD_2_, ΔD_98_, and ΔTV_PIV_. Considering that the dosimetric accuracy without daily CBCT could be impaired with increasing body changes and corresponding setup errors, CI_body_ and ΔV_n_ could be used as an indicator for clinical radiation therapists to provide feedback to physicists for assessments of CBCT scheduling and replanning in clinical treatment.

## Data Availability

The original contributions presented in the study are included in the article/supplementary material. Further inquiries can be directed to the corresponding author.
